# TRPML1 and RAS-driven cancers – exploring a link with great therapeutic potential

**DOI:** 10.1080/19336950.2019.1666457

**Published:** 2019-09-17

**Authors:** Jewon Jung, Kartik Venkatachalam

**Affiliations:** a Department of Integrative Biology and Pharmacology, McGovern Medical School at the University of Texas Health Sciences Center (UTHealth), Houston, TX, USA; b Graduate Program in Biochemistry and Cell Biology, MD Anderson Cancer Center and UTHealth Graduate School of Biomedical Sciences, Houston, TX, USA

**Keywords:** Endolysosomes, TRPML1, lysosomes, mucolipins, HRAS, RAS, cancer, cholesterol, TFEB

## Abstract

Activating mutations in the *RAS* family of proto-oncogenes represent some of the leading causes of cancer. Unmitigated proliferation of cells harboring oncogenic *RAS* mutations is accompanied by a massive increase in cellular bioenergetic demands, which offers unique opportunities for therapeutic intervention. To withstand the steep requirements for metabolic intermediates, RAS-driven cancer cells enhance endolysosome and autophagosome biogenesis. By degrading cellular macromolecules into metabolites that can be used by biosynthetic pathways, endolysosomes permit continued proliferation and survival in otherwise detrimental conditions. We recently showed that human cancers with activating mutations in *HRAS* elevate the expression of *MCOLN1*, which encodes an endolysosomal cation channel called TRPML1. Increased TRPML1 activity in HRAS-driven cancer cells is needed for the restoration of plasma membrane cholesterol that gets collaterally internalized during endocytosis. Inhibition of TRPML1 or knockdown of *MCOLN1* leads to mislocalization of cholesterol from the plasma membrane to endolysosomes, loss of oncogenic HRAS from the cell surface, and attenuation of downstream signaling. Here, we discuss the implications of our findings and suggest strategies to leverage pathways that impinge upon TRPML1 as novel anti-cancer treatments.

## Introduction

KRAS, HRAS, and NRAS are small GTPases encoded by an evolutionarily conserved family of proto-oncogenes [1,2]. These fascinating proteins operate at the nexus of growth factor receptors and mitogen-activated protein kinase (MAPK) cascades, and are responsible for the faithful transmission of signals between the two [1–3]. Over 25% of human tumors harbor “oncogenic” (i.e. activating) mutations in *RAS* genes [4–9], which makes their protein products some of the most important therapeutic targets in cancer [10–16]. In healthy cells, cell surface receptors are functionally coupled to RAS proteins, which incite phosphorylation cascades involving RAF–MEK–ERK or phosphatidylinositol-3-kinase (PI3K) [1–3]. Phosphorylated ERK, the activated form of a terminal MAPK, translocates to the nucleus where it induces the expression of growth-related genes [1–3,14]. Oncogenic mutations usually abolish the intrinsic GTPase activity of RAS and lock the proteins in GTP-bound constitutively active state. As a result, ERK phosphorylation and attendant cell proliferation are dramatically higher in cells harboring these mutations [4–6,11].

Despite more than three decades of concerted effort, effective anti-RAS therapies have remained elusive. The paucity of success has even prompted the notion that RAS proteins might be “undruggable” [12,13,15]. Although this idea is now being challenged by new classes of drugs [17], a traditional approach to sidestep the need to target RAS directly focused on inhibition of downstream kinases [11,13,16,18,19]. Unfortunately, these strategies were ultimately ineffective due to intractable feedback loops and the propensity for acquired resistance [18,20]. A good example is the pharmacological inhibition of BRAF, which induces paradoxical activation of RAS–ERK signaling and undesirable potentiation of cell proliferation [18,20]. Another cogent strategy to tune-down the proliferative effects of mutant RAS relies on the identification of orthogonal cellular pathways that make hyperactive RAS–ERK signaling possible. Genomic, proteomic, and other modern analytical techniques have led to the identification of pathways that are potentiated in cancers. In this arena, lysosomal proteins are emerging as an attractive group that can be targeted to mitigate tumorigenesis [21–30]. Owing to their roles in cellular metabolism, intracellular trafficking, and macromolecule recycling, lysosomes sustain hallmarks of cancer – abnormal proliferation, drug resistance, metastasis, and angiogenesis [28–34]. Based on this understanding, it has been asserted that the disruption of endolysosomal function can retard the growth of certain malignancies.

## Dysregulated lysosomal biogenesis in cancer cells

**Figure 1.**
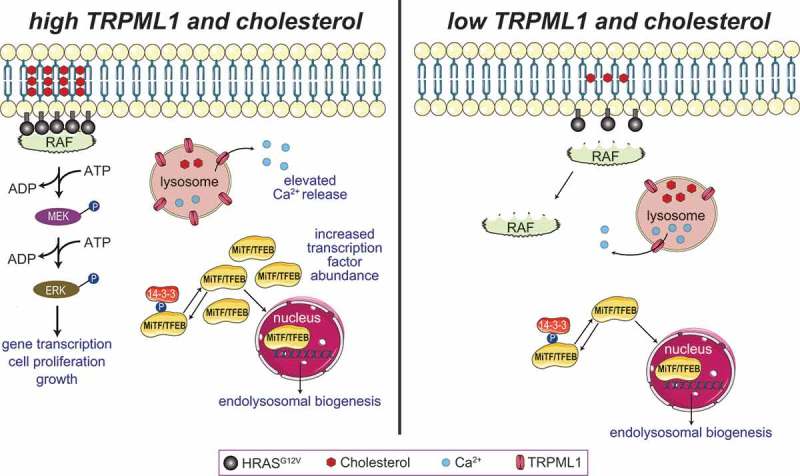
Role of TRPML1 in the regulation of ERK signaling in cells harboring *HRAS^G12V^* mutations. HRAS–ERK signaling involves the assembly of HRAS monomers into nanodomains that can recruit RAF, and promote MEK and ERK phosphorylation (*left*). HRAS^G12V^ nanoclusters formation and plasma membrane localization are predicated upon the availability of cholesterol. When plasma membrane cholesterol is depleted or lowered (*right*), HRAS^G12V^ monomers cannot recruit RAF, leading to diminished ERK phosphorylation. Cancer cells harboring oncogenic HRAS showed an increased level of MiTF/TFE transcriptional factors and activation of the CLEAR gene network that leads to the lysosomal biogenesis (*left*). The elevated level of TRPML1 results in increased lysosomal Ca^2+^ release, HRAS^G12V^ nanoclusters in the plasma membrane, activation of MAPK pathway, and cell proliferation.

Cancer cell proliferation, which requires sustained biosynthesis of a variety of macromolecules, imposes a massive requirement for nutrients. To cope with steep metabolic demands, cancer cells generate copious numbers of autophagosomes and endolysosomes, which break down cellular macromolecules to intermediates that are shunted toward growth [21,23]. Leveraging a transcriptional pathway necessary for autophagy and endolysosomal biogenesis [35–38], cancer cells upregulate several endolysosomal proteins and enzymes *en masse* [21,23]. The bHLH transcription factors – transcription factor EB (TFEB), transcription factor binding to IGHM enhancer 3 (TFE3), and melanocyte inducing transcription factor (MiTF) – drive the expression of many genes that encode endolysosomal proteins [38–42]. Not surprisingly, the activities of these transcription factors are coupled to nutrient availability. For instance, under nutrient-rich conditions, TFEB is phosphorylated by the mechanistic target of rapamycin complex 1 (mTORC1) and sequestered in the cytoplasm by 14-3-3 protein [39,42,43] (Figure 1). When nutrient levels drop, mTORC1 activity declines [44]. Consequently, TFEB is dephosphorylated and translocates to the nucleus, where it activates the CLEAR (coordinated lysosomal expression and regulation) gene network leading to enhanced lysosomal biogenesis [35,39,40,42] (Figure 1). Along with the emergence of the understanding that lysosomal biogenesis is a potential therapeutic avenue, elevated TFEB, MiTF, and TFE3 activities have been described in various malignancies [23,27,29,41,45,46]. In KRAS-driven pancreatic ductal adenocarcinoma (PDAC), TFEB is decoupled from mTORC1, and constitutively translocates to nuclei where it compels endolysosomal biogenesis [23,47]. In other instances, cancer-related mutations affect the expression of these transcription factors. For instance, genomic translocations in renal cell carcinoma and soft tissue sarcoma lead to *TFEB* and *TFE3* overexpression, whereas *MiTF* expression is increased in melanoma and hepatocellular carcinomas [41,48–52].

When thinking about translating these insights into feasible therapies, we are faced with numerous challenges. In addition to mediating endolysosomal biogenesis, TFEB, MiTF, and TFE3 are also involved in biological processes that are (at least ostensibly) unrelated to endolysosomal function. For example, MiTF is required for the specification of melanocytes [52,53]. Furthermore, some cancer cells simultaneously upregulate two or more of these proteins [29]. In this situation, inhibition of one transcription factor might not be sufficient for adequate attenuation of endolysosomal biogenesis. To make matters worse, many genes that encode non-lysosomal proteins also possess the CLEAR motif and are likely regulated by TFEB, TFE3, and MiTF. This makes the onset of unintended consequences a distinct possibility in cells treated with putative inhibitors of these transcription factors. The CLEAR motif that is bound by TFEB, TFE3, and MiTF is identical to the canonical E-box that is the target of many different bHLH transcription factors including Myc and Max [36,54,55]. Thus, Myc/Max and other bHLH transcription factors likely influence the effects of TFEB, MiTF and TFE3 to drive endolysosomal biogenesis in certain cancers. Supporting this idea, a recent study showed that Myc competes with TFEB and TFE3 for binding to CLEAR elements and thus negatively influences lysosomal biogenesis [56]. To overcome these limitations, we reasoned that targeting endolysosomal proteins that play tumor-specific roles might be a better strategy to attenuate tumor growth. The question we are faced with now is – *which endolysosomal proteins should we go after?*

## Involvement of a lysosomal Ca^2+^ channel, TRPML1, in the proliferation of cells harboring oncogenic HRAS mutations

We sought to identify endolysosomal proteins that could be targeted to mitigate the growth of cancer cells. Using a genomic approach and oncogenic HRAS-driven cancer cells as models, we identified the endolysosomal Ca^2+^ permeable channel, TRPML1, as a potential target in cells harboring oncogenic *HRAS* [29]. Using datasets available at the cancer genome atlas (TCGA), we found that the gene encoding the endolysosomal cation channel, TRPML1 (*MCOLN1*), was upregulated in oncogenic *HRAS*-expressing tumors due to the combined actions of MiTF and TFEB. Accompanying the increase in *MCOLN1* expression, TRPML1-mediated endolysosomal Ca^2+^ release was dramatically higher in HRAS-driven cancer cells compared to controls (Figure 1). Importantly, elevated expression of *MCOLN1* in patients with HRAS-positive tumors correlated with poorer prognosis [29]. We went on to show that genetic or pharmacological inhibition of TRPML1 reduced the proliferation of many different oncogenic HRAS-expressing cancer cell lines via attenuation of the MEK–ERK pathway (Figure 1). TRPML1 activity depends on the vesicular phosphoinositide, PI(3,5)P_2_, which is synthesized by the PIK-FYVE (FYVE-containing phosphoinositide kinase) lipid kinase complex containing Fab1 and Vac14 [57,58]. Pharmacological inhibition of PIK-FYVE or knockdown of *VAC14* also selectively inhibited the proliferation of mutant HRAS-driven cancer cells [29]. Interestingly, *MCOLN1* knockdown or TRPML1 inhibition did not affect ERK phosphorylation and cell proliferation in cancer cells expressing wild-type *HRAS*, or in cells in which oncogenic *HRAS* was stably knocked down [29]. These data indicate that inhibition of TRPML1 imparts selective vulnerability to cells expressing oncogenic HRAS while leaving cells with normal HRAS unaffected. Thus, TRPML1 appears generally dispensable for regulating the gain of MAPK signaling, except in the context of HRAS-driven cancer cells that are marked by the profound upregulation of ERK phosphorylation. If these phenotypes were to be reproduced in the clinic, one could speculate selective attenuation of cancer cell proliferation with diminished side effects associated with perturbation of otherwise healthy cells.

The utility of blocking TRPML1 was also observed in vertebrate xenograft models and *Drosophila* lacking the TRPML1 homolog [29,59]. These findings paint a picture of an evolutionarily conserved pathway of endolysosomal Ca^2+^ release that is required for elevated RAS activity. Attenuation of cell proliferation and RAS signaling in TRPML-deficient *Drosophila* expressing *Drosophila Ras^G12V^* or human *HRAS^G12V^* also demonstrate a fundamental requirement for the vesicular channels that extends beyond the genetic “ecosystem” of cancer cells [29]. This was important from a conceptual standpoint since cancer cells carry hundreds of mutations that synergistically drive proliferation and survival [60].

**Figure 2.**
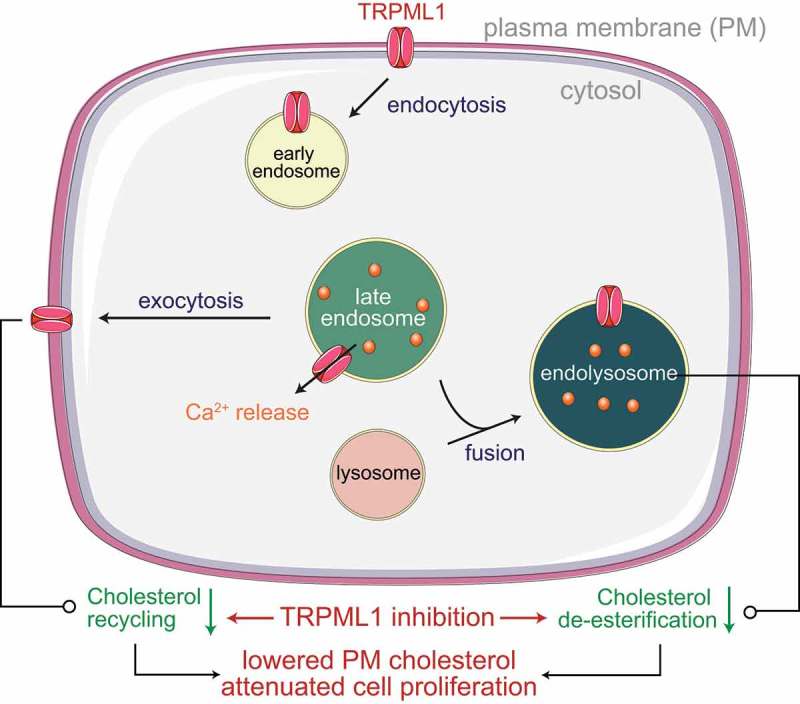
TRPML1 is necessary for vesicular trafficking and fusion of endolysosomes with lysosomes or the plasma membrane (PM). By mediating endolysosomal exocytosis, TRPML1 coordinates the restoration of PM lipids such as cholesterol that get collaterally internalized during endocytosis. Delivery of endocytosed material to lysosomes is needed for de-esterification of cholesterol esters. Defects in de-esterification and recycling lead to lowered PM cholesterol, which explains the attenuation of HRAS^G12V^-driven ERK signaling.

*How does an endolysosomal cation channel regulate HRAS–ERK signaling in cancer cells?* Localization and clustering of HRAS at the plasma membrane, which is dependent on cholesterol levels, is required for the engagement of downstream effectors (Figure 1) [2,3,61–64]. Given the involvement of TRPML1 in vesicle exocytosis (Figure 2), *MCOLN1* knockdown or TRPML1 inhibition diminished the movement of cholesterol from endolysosomal vesicles to the plasma membrane [29,65–69]. Accompanying these defects in cholesterol recycling, inhibition of TRPML1 also prevented the de-esterification of endocytosed cholesterol esters (Figure 2) [29]. Consequently, levels of plasma membrane cholesterol fell to an extent that was sufficient to disrupt HRAS^G12V^ nanocluster formation and plasma membrane abundance, and thereby, attenuate ERK phosphorylation and cell proliferation (Figure 1). Nanoclusters of wild-type HRAS were also perturbed by TRPML1 inhibition, but the abundance of this variant in the plasma membrane was not altered. These data explain the heightened sensitivity of HRAS^G12V^ to TRPML1 inhibition. Further supporting the involvement of cholesterol, lowering cholesterol levels by application of statins phenocopied TRPML1 inhibition, whereas supplementation of cholesterol prevented the effects of TRPML1 inhibition [29].

## Conclusions and future perspectives

Our studies raise the intriguing possibility that targeting TRPML1 function might be a viable therapeutic option for HRAS-driven cancers. The notion of inhibiting TRPML1 to attenuate tumor growth agrees with findings of *MCOLN1* upregulation and a requirement for TRPML1 in proliferation and metastases of triple-negative breast cancer cells, and the survival/proliferation of melanoma cells [28,30]. Taken together, these three studies highlight some of the complexities of the relationship between TRPML1 and cancer. Similar to our findings in HRAS-driven cancer cells, Xu et al. demonstrate that TRPML1 promotes the development of triple-negative breast cancer via lysosomal exocytosis and the attendant release of ATP [25]. TRPML1 also positively regulates mTORC1 activity, and this function of the channel further promotes the breast cancer cell proliferation [25]. In a departure from this theme, TRPML1 negatively regulates both ERK phosphorylation and mTORC1 activity in melanoma [28]. Rather, the utility of blocking TRPML1 in melanoma stems from a role for the channel in potentiation of micropinocytosis [28]. As suggested by Kasitinon et al. [28], differences in the purported mechanisms by which TRPML1 promotes tumorigenesis likely reflects the presence of distinct driver mutations in the different types of cancer. If so, the qualitative similarities of the effects of TRPML1 inhibition in all three cancers are even more remarkable. The idea that endolysosomal Ca^2+^ release promotes tumorigenesis likely extends to other modes of cation release from these organelles [70,71]. For instance, Ca^2+^ permeable, nonselective endolysosomal two-pore cation channels were found to play an important role in cancer cell migration via β1-integrin trafficking and recycling [72]. Additionally, the TRPML1 paralog, TRPML2, has been shown to be involved in the onset of glioma, and inhibitors of PI(3,5)P_2_ biosynthesis are effective in the attenuation of cell proliferation in hepatic and hematological malignancies [73–75]. Together, these findings promise an era of cancer therapeutics that revolve around endolysosomal ionic homeostasis.

Despite our excitement, additional studies are going to be needed before we head to the clinic. Loss-of-function mutations in *MCOLN1* are responsible for a severe childhood-onset lysosomal storage disease called mucolipidosis type IV (MLIV) [76–78]. Thus, the onset of potentially severe neurological dysfunction could deter the administration of TRPML1 inhibitors to humans. That being said, neurological deficits are unlikely to be a major barrier if the drug being administered is unable to inhibit the channel in the nervous system. One could consider using antisense oligonucleotides (ASOs) to diminish *MCOLN1* expression in cancers. Given that ASOs are inherently unable to cross the blood–brain barrier [79], these drugs bear the potential to attenuate endolysosomal Ca^2+^ release in non-neuronal tumors while leaving *MCOLN1* expression unchanged in the CNS. These and other possibilities ensure our ongoing enthusiasm regarding the development of new anti-cancer strategies that leverage the function of endolysosomal ion channels.
